# Of the Good Effects of a Decoction of the Outer Shell of the Walnut in the Cure of Ulcers

**Published:** 1789

**Authors:** J. Hunczousky

**Affiliations:** Professor of the Operations of Surgery, &c. in the Imperial and Royal Josephine Medico-Chirurgical Academy at Vienna


VII. *Of the good Effects of a Decoction of the outer Shell of the Walnut in the Cure of Ulcers. By* J. Hunczousky, *Professor of the Operations of Surgery, &c. in the Imperial and Royal Josephine Medico-Chirurgical Academy at Vienna.* Vide *Acta Academiæ Cæs. Reg. Josephinæ Medico-Chirurgicæ Vindobonensis* *, Tom. I. 4to. Vindobonæ, 1788.

THE decoction here recommended, as a topical remedy in ulcers, is prepared by macerating an ounce of the dried green outer

* Besides this edition, in Latin, of their Transactions, the Academy have published another in German, which we have already announced. See Vol. IX. page 101.

shell,

ſhell, or huſk, of the walnut in a pint of water during three or four hours, and afterwards boiling theſe ingredients for about a quarter of an hour. The liquor, when cold, is to be filtered; and lint moiſtened with it is to be applied to the ſores.

The author mentions the good effects of this application in ulcers in general; but more particularly in what he calls broad, flaccid ulcers *, and in moiſt, herpetic ulcers, which he ſuppoſes to originate from a local acrimony in the ſkin, and which are not attended with inflammation †.

He aſſerts, that by means of this remedy the healing of the ulcer is promoted with a degree of celerity that is ſeldom experienced from other applications; and that during its employment the uſe of eſcharotics is never neceſſary for the removal of ſpongy fleſh, as the decoction never fails to remove this fungus in the courſe of a few days.

A particular account is given of ſeven caſes in which he has ſucceeded with this decoction

* Ulcera lata et flaccida.

† Ulcera humida, herpetica, quæ originem ſuam a quadam acrimonia in cute ſita trahunt, et ubi nulla inflammatio adeſt.

after after a variety of other topical applications (though assisted by purgative and other internal remedies) had proved ineffectual; and the author adds, that he has found it equally serviceable in upwards of thirty other cases of ulcers in which he has tried it, and in which the usual remedies had failed. †

Whatever may be the merits of this application, it cannot be said to be altogether new; for we find a preparation very similar to it, viz. a decoction of the leaves of the walnut tree, with the addition of a little sugar, recommended by Belloste as a topical remedy of singular efficacy in ulcers.

Belloste observes, that many persons in France had made a great secret of this decoction; but that he should have thought himself deficient in humanity had he omitted to make known its good qualities and the manner of preparing it. He assures us that in a thousand instances he had experienced its good effects; and that, since he had been in the habit of employing it, ulcers, till then considered as incurable, had been healed by it in a very short space of time *.

* See his work, entitled *Le Chirurgien d'Hopital.* 4th Edition. 8vo. Amsterdam, 1707. p. 239.

